# Regional Disparities in the Prevalence of Probing Depths and Tooth Loss in Germany—Results From the German National Cohort (NAKO)

**DOI:** 10.1111/jcpe.70201

**Published:** 2026-09-18

**Authors:** Jana Gortschakow, Katrin Borof, Dominic Sasunna, Kathrin Kuhr, Ghazal Aarabi, Sebastian‐Edgar Baumeister, Antonio Ciardo, Karin Halina Greiser, Daniel Hagenfeld, Katrin Hertrampf, Ti‐Sun Kim, Jan Kühnisch, Tobias Pischon, Stefan L. Reckelkamm, Stefanie Samietz, Martin Wojciechowsky, Asma'a Alselwi, Yvonne Kemmling, Lilian Krist, Michael Leitzmann, Claudia Meinke‐Franze, Rafael Mikolajczyk, Nadia Obi, Regina Pickford, Nicole Pischon, Wolfgang Rathmann, Matthias B. Schulze, Sabine Schipf, Börge Schmidt, Stefan N. Willich, Stefan Rach, A. Rainer Jordan, Henry Völzke, Thomas Kocher, Philipp Kanzow, Alexander Welk, Stefan Rupf, Birte Holtfreter

**Affiliations:** ^1^ Department of Restorative Dentistry, Periodontology and Endodontology University Medicine Greifswald Greifswald Germany; ^2^ Department of Periodontics, Preventive and Restorative Dentistry, Centre for Dental and Oral Medicine University Medical Centre Hamburg‐Eppendorf Hamburg Germany; ^3^ Institut der Deutschen Zahnärzte (IDZ) Cologne Germany; ^4^ Institute of Health Services Research in Dentistry University of Münster Münster Germany; ^5^ Section of Periodontology, Department of Conservative Dentistry, Clinic for Oral, Dental and Maxillofacial Diseases, Heidelberg Faculty of Medicine Heidelberg University Heidelberg Germany; ^6^ Division of Cancer Epidemiology German Cancer Research Center (DKFZ) Heidelberg Germany; ^7^ Clinic for Periodontology and Conservative Dentistry University of Münster Münster Germany; ^8^ Department of Oral and Maxillofacial Surgery University Hospital Schleswig‐Holstein Kiel Germany; ^9^ Center of Health Services Research in Dentistry University Hospital Schleswig‐Holstein Kiel Germany; ^10^ Department of Conservative Dentistry and Periodontology, Ludwig‐Maximilian University Hospital Ludwig‐Maximilian University Munich Munich Germany; ^11^ Max Delbrück Center for Molecular Medicine in the Helmholtz Association (MDC), Molecular Epidemiology Research Group Berlin Germany; ^12^ Charité – Universitätsmedizin Berlin, Corporate Member of Freie Universität Berlin and Humboldt‐Universität Zu Berlin Berlin Germany; ^13^ Department of Prosthetic Dentistry, Gerodontology and Biomaterials University Medicine Greifswald Greifswald Germany; ^14^ Institute of Epidemiology Helmholtz Zentrum München, German Research Center for Environmental Health Neuherberg Germany; ^15^ Medical Faculty, Chair of Epidemiology, Institute for Medical Information Processing, Biometry and Epidemiology Ludwig‐Maximilians‐University Munich Germany; ^16^ Department of Epidemiology Helmholtz Centre for Infection Research (HZI) Braunschweig Germany; ^17^ Institute of Social Medicine, Epidemiology and Health Economics, Charité – Universitätsmedizin Berlin Berlin Germany; ^18^ Department of Epidemiology and Preventive Medicine University of Regensburg Regensburg Germany; ^19^ Department for the Study of Health in Pomerania (SHIP) and Clinical Epidemiology, Institute for Community Medicine University Medicine Greifswald Greifswald Germany; ^20^ Institute for Medical Epidemiology, Biometrics, and Informatics (IMEBI), Interdisciplinary Center for Health Sciences Medical Faculty of the Martin Luther University Halle‐Wittenberg Halle Germany; ^21^ Institute for Occupational and Maritime Medicine (ZfAM) University Medical Center Hamburg‐Eppendorf Hamburg Germany; ^22^ Zahn‐ und Prophylaxe Centre Priv.‐Doz. Dr. Nicole Pischon Berlin, Schönefeld Germany; ^23^ Institute for Biometrics and Epidemiology, German Diabetes Center, Leibniz Center for Diabetes Research at Heinrich Heine University Düsseldorf Düsseldorf Germany; ^24^ Department of Molecular Epidemiology German Institute of Human Nutrition Potsdam‐Rehbruecke Nuthetal Germany; ^25^ Institute of Nutritional Science University of Potsdam Nuthetal Germany; ^26^ Institute for Medical Informatics, Biometry and Epidemiology, University Hospital of Essen University of Duisburg‐Essen Duisburg Germany; ^27^ Department Epidemiological Methods and Etiological Research Leibniz‐Institute for Prevention Research and Epidemiology – BIPS Bremen Germany; ^28^ Synoptic Dentistry Saarland University Homburg/Saar Germany

**Keywords:** Cohort study, edentulism, German national cohort, NAKO, periodontitis, prevalence, regional disparities

## Abstract

**Aim:**

To evaluate regional disparities in the prevalence, severity and extent of probing depths (PDs) as well as tooth loss in German adults aged 19–74, using data from the baseline study of the German National Cohort (NAKO; 2014–2019).

**Methods:**

Dental examinations were recorded at two levels: Level 1 (18/18 centres; *N* = 184,871; tooth counts including third molars and dental interview) and Level 2 (8/18 centres, not geographically representative; *N* = 18,389; recording of PDs). We evaluated differences by sex, age and study region. All data were survey‐weighted. For individuals aged 65–74, we supplemented tooth loss data from the Fifth German Oral Health Study and the Study of Health in Pomerania.

**Results:**

Among Level 1 participants, the prevalence of edentulism was 1.7% (95% confidence interval: 1.6–1.9). The average mean PD was 1.89 mm (1.87–1.91). Both edentulism and PDs increased with increasing age. There were clear regional disparities, with the lowest prevalence of both in Augsburg/Regensburg in the south (1.3% and 1.64 mm) and the highest in Neubrandenburg in the north‐east (2.3% and 2.31 mm).

**Conclusion:**

Clear regional disparities emerged. In light of demographic changes, these results highlight the importance of incorporating region‐specific oral health burdens into future healthcare planning and treatment concepts.

## 1 Introduction

Periodontitis is a chronic inflammatory disease characterised by the progressive destruction of the tooth‐supporting structures (Sanz et al. 2020). It is a highly prevalent condition, with over 1 billion cases worldwide in 2021 (Nascimento et al. 2024). Unchanged high‐level prevalences were also reported for Germany, and the proportion of undiagnosed and untreated cases remains high (Eickholz et al. 2025).

Ongoing demographic changes and an ageing population will presumably have a significant impact on the burden of periodontitis and its risk factors (Tonetti et al. 2017). With people retaining more of their teeth for longer, more are exposed to periodontitis, leading to a greater need for treatment. As periodontitis often presents with few or mild symptoms in the initial stages, it can be overlooked and not diagnosed at all, resulting in delayed treatment initiation at a more advanced stage, reduced treatment success and worse outcomes for the remaining teeth. Consequently, as treatment costs increase with disease severity, the economic burden of periodontitis is likely to rise, including direct, indirect and intangible costs (Pattamatta et al. 2025).

While the German Oral Health Studies (DMS) provided valuable insights into national trends in the prevalence and severity of periodontitis (Kocher, Eickholz, et al. 2025), detailed regional analyses and comparisons are lacking. However, given the heterogeneous distribution of risk factors (Michalski et al. 2022), it is reasonable to assume that such differences exist. For instance, the prevalence of Type 2 diabetes mellitus is still more prevalent in eastern and north‐eastern Germany than in southern Germany (Schipf et al. 2014). Furthermore, disparities in social life and deprivation resulting from the historical development of the healthcare systems in the former German Democratic Republic and the former Federal Republic of Germany were still evident during the German National Cohort's (NAKO) baseline examinations.

Detailed regional analyses are crucial for the future development of regionally tailored health services and optimal resource allocation for the treatment of periodontitis. In this study, therefore, we examined the prevalence, severity and extent of probing depths (PDs) as well as the prevalence of edentulism and number of present teeth at the regional level using data from NAKO. To contextualise regional patterns among older adults aged 65–74 years, tooth loss data from two additional population‐based German studies with similar examination periods were included: the Fifth German Oral Health Study (DMS V; 2013–2014); and the Study of Health in Pomerania (SHIP‐TREND‐0; 2008–2012).

## 2 Materials and Methods

### 2.1 German National Cohort (NAKO)

#### 2.1.1 Study Design

NAKO is a large‐scale, multidisciplinary, nationwide, multi‐centre, population‐based, prospective cohort study (Peters et al. 2022). Briefly, the study recruited over 200,000 participants aged 20–69 years from the general population living in the adjacent areas of 18 study centres (see Figure S1) using samples drawn randomly from municipal registries. A sex‐ and age‐stratified sampling design was used, with equal balance among sexes, with 10.0% of participants in each 10‐year age group from 20 to 39 and 26.7% in each 10‐year age group from 40 to 69. The study's methodology is designed to cover a wide array of urban, industrial and rural settings throughout the country, thereby ensuring a diverse demographic spread. Data collection for the baseline examination (2014–2019) included 205,415 participants aged 19–74 years at the time of examination. Response was 15.6%, with study‐region‐specific responses ranging from 7.6% in Berlin‐Süd to 30.7% in Augsburg (Rach et al. 2025).

Supporting Information provides detailed information about the study design (Level 1 and Level 2 examinations), the Level 1 and Level 2 dental and periodontal examinations as well as information about reliability data and covariates.

### 2.2 DMS V and SHIP‐TREND‐0

Supporting Information provides detailed information about the study design, dental examinations, reliability studies and covariates for the DMS V and SHIP‐TREND‐0 studies.

### 2.3 Statistical Analyses

The presence of edentulism (i.e., the absence of all natural teeth) was derived in all studies. A dentate person is someone who has at least one natural tooth. In NAKO, edentulism was determined using Level 1 data on tooth counts (full‐mouth, including third molars). From PD data, the prevalence, the percentage of affected sites, the number of affected teeth and the percentage of affected teeth were determined for two thresholds, that is, PD ≥ 4 mm and PD ≥ 6 mm. Prevalences were determined according to two ways: (i) including edentulous individuals in the denominator (edentulous individuals were counted as non‐prevalent individuals; information from Level 1 tooth counts was considered to define edentulism); and (ii) excluding edentulous individuals.

The characteristics of the study populations were described by means of mean values and standard deviations (SDs) for continuous variables, and absolute and relative frequency distributions for categorical variables.

Firstly, we presented tooth loss and interview data (Level 1) and PD data (Level 2) for NAKO, reporting means with 95% confidence intervals (CI) in total and according to sex, age and study region/cluster (Level 1) or study region (Level 2). Data were also graphically presented by plotting means and the corresponding 95% CIs. Because of the complex sample design, standard errors (SEs) were calculated considering survey weights. Survey weights were specifically determined for this project using a three‐step procedure (Rach et al. 2025), multiplicatively combining design weights, propensity score weights and calibration weights. While design weights only accounted for the sampling procedure or the selection probability of survey units, calibration weights accounted for differential non‐response and the distribution of certain characteristics within the population (age group, sex, nationality, education, migration background and household size). Propensity score weights accounted for selection differences between the group that completed the Level 2 examinations and the group that did not. Missing values were not imputed. While interval estimation was used to quantify effect sizes and their precision, formal pairwise hypothesis testing was not conducted.

Secondly, we examined the association between the study region and (i) the prevalence of PD ≥ 4 mm (modified Poisson regression; robust standard errors; incidence rate ratios with 95% CIs), and (ii) the mean PD (generalised linear models with a gamma distribution and a log link; effect estimates expressed as a mean ratio [MR]) (Manning et al. 2005), using both complete case and multiply imputed data (chained equations with 10 imputations; including age, sex, mean PD and study region). The following factors were included in a stepwise manner: (i) age (10‐year categories) and sex, and (ii) plus education, smoking status and diabetes mellitus. Thirdly, we separately analysed tooth loss data for individuals aged 65–74 from the DMS V and SHIP‐TREND‐0. Survey weighted estimates were reported.

Statistical significance was set at *p* < 0.05. All analyses were performed using Stata/SE Version 17.0 (StataCorp 2021) and R 4.3.3 (R Core Team 2025). This article is written in accordance with recommendations issued by the ‘Strengthening the Reporting of Observational Studies in Epidemiology (STROBE)’ initiative (von Elm et al. 2007).

## 3 Results

### 3.1 NAKO Baseline Characteristics

The average age of Level 1 participants (*N* = 184,871) was 49.6 years, and 49.7% were male (Table 1). For Level 2 participants (*N* = 18,389), the average age was 49.7 years, and 52.4% were male. Approximately half of the participants were current or former smokers. Around 60% reported at least 14 years of education. With regard to the study region, the highest prevalences of current smoking and diabetes were observed in Halle/Leipzig and Neubrandenburg, and the lowest prevalences for ≥ 14 years of education were found in Augsburg/Regensburg and Neubrandenburg (Tables S1 and S2).

**TABLE 1:** Characteristics of Level 1 and Level 2 participants of NAKO.

	Level 1 (*N* = 184,871)	Level 2 (*N* = 18,389)
Age, years	49.6 ± 12.8	49.7 ± 12.4
Sex		
Male	91,843 (49.7%)	9633 (52.4%)
Female	93,028 (50.3%)	8756 (47.6%)
Smoking status		
Never	82,743 (44.8%)	8660 (47.1%)
Former	59,096 (32.0%)	5882 (32.0%)
Current	35,748 (19.3%)	3416 (18.6%)
Missing	7284 (3.9%)	431 (2.3%)
Years of theoretical education^a^		
≤ 10 years	4552 (2.5%)	353 (1.9%)
11–13 years	47,691 (25.8%)	4938 (26.8%)
14–17 years	75,334 (40.7%)	8006 (43.6%)
≥ 18 years	40,542 (21.9%)	3487 (19.0%)
Missing	16,752 (9.1%)	1605 (8.7%)
Diabetes mellitus		
No	173,727 (94.0%)	17,457 (94.9%)
Yes	10,701 (5.8%)	902 (4.9%)
Missing	443 (0.2%)	30 (0.2%)
Dental visit within the last 12 months		
No	18,092 (9.8%)	1713 (9.3%)
Yes	117,102 (63.3%)	11,737 (63.8%)
Missing	49,677 (26.9%)	4939 (26.9%)

*Note:* Data are presented as unweighted means ± standard deviation or *N* (%).

^a^ International Standard Classification of Education 97 (ISCED‐97).

### 3.2 Prevalence of Edentulism and Number of Teeth (Level 1)

Among Level 1 participants, the prevalence of edentulism was 1.7% (Table 2). It was the same for both males and females (1.7%) and increased from 0.04% in the youngest age group to 5.9% in the oldest. Actual differences were all marginal for edentulism (all < 5%) but clinically relevant for the number of teeth in dentates (i.e., ≥ 1 in at least one pairwise comparison). The Augsburg/Regensburg region had the best results (edentulism at 1.3%; 25.9 teeth in dentates), while Neubrandenburg (edentulism at 2.3%; 24.1 teeth in dentates) and the other northern study regions (Kiel and Hannover/Hamburg/Bremen) had the worst results (Figure 1).

**TABLE 2:** Overview on prevalence of edentulism, the number of teeth and the number of teeth in dentates using Level 1 data from NAKO. Data are presented in total and stratified by sex, age and study region (*N* = 184,871).

	Unweighted number of subjects	Edentulism, %	Number of teeth	Unweighted number of dentates	Number of teeth in dentates
Total	184,871	1.7 (1.6–1.9)	25.3 (25.3–25.4)	182,422	25.8 (25.7–25.8)
By sex					
Female	93,028	1.7 (1.6–1.9)	25.0 (25.0–25.1)	91,924	25.5 (25.4–25.5)
Male	91,843	1.7 (1.6–1.9)	25.7 (25.6–25.7)	90,498	26.1 (26.0–26.2)
By age					
19–29 years	18,603	0.04 (0.0–0.1)	28.6 (28.5–28.6)	18,599	28.6 (28.5–28.6)
30–39 years	19,932	0.1 (0.04–0.3)	28.0 (27.9–28.1)	19,926	28.0 (28.0–28.1)
40–49 years	48,582	0.4 (0.3–0.6)	26.6 (26.5–26.6)	48,509	26.7 (26.6–26.7)
50–59 years	49,292	2.1 (1.8–2.4)	23.9 (23.8–24.0)	48,746	24.4 (24.3–24.5)
60–74 years	48,462	5.9 (5.4–6.3)	20.1 (20.0–20.3)	46,642	21.4 (21.2–21.5)
By study region					
Augsburg/Regensburg	27,791	1.3 (1.0–1.7)	25.5 (25.4–25.7)	27,452	25.9 (25.8–26.0)
Mannheim/Freiburg/Saarbrücken	27,088	1.7 (1.4–2.0)	25.6 (25.5–25.8)	26,768	26.0 (25.9–26.2)
Essen/Muenster/Duesseldorf	28,189	1.6 (1.4–1.9)	25.6 (25.5–25.7)	27,810	26.0 (25.9–26.1)
Halle/Leipzig	17,138	1.5 (1.3–1.9)	25.3 (25.1–25.4)	16,900	25.7 (25.5–25.8)
Berlin‐Süd/Mitte/Nord	29,592	1.7 (1.5–2.0)	25.7 (25.5–25.8)	29,191	26.1 (26.0–26.2)
Hann‐over/Hamburg/Bremen	28,612	2.0 (1.7–2.3)	25.5 (25.4–25.7)	28,236	26.0 (25.9–26.1)
Kiel	8861	2.1 (1.6–2.8)	24.9 (24.7–25.2)	8757	25.5 (25.2–25.7)
Neubrandenburg	17,600	2.3 (1.9–2.8)	23.6 (23.4–23.8)	17,308	24.1 (24.0–24.3)

*Note:* Data are presented as survey‐weighted estimates with 95% confidence intervals.

**FIGURE 1:**
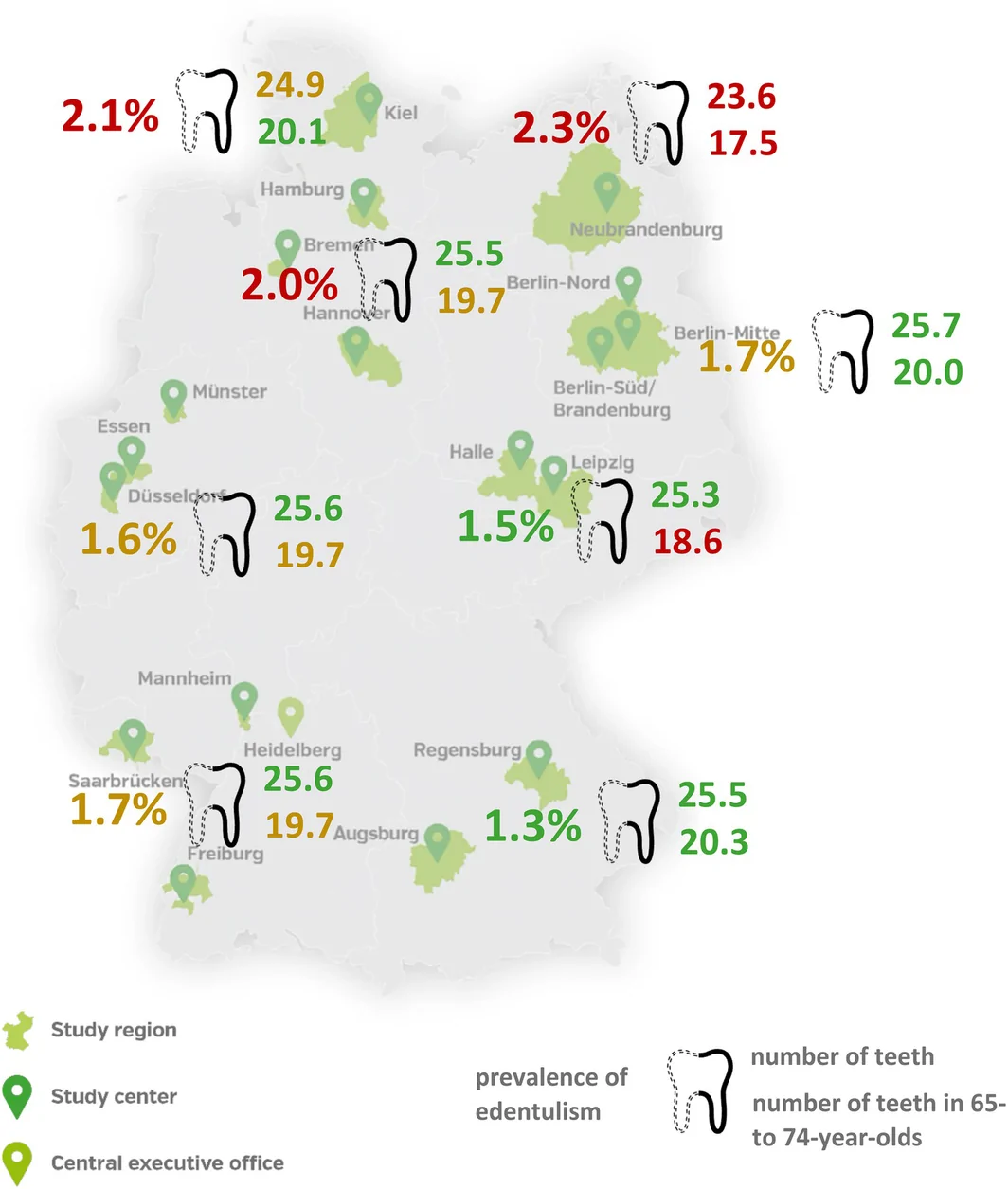
Coloured map showing prevalences of edentulism, the number of teeth and the number of teeth in participants aged 65–74 for each of the Level 1 study regions of NAKO.

### 3.3 Prevalence, Severity and Extent of Probing Depths (Level 2)

On including edentulous individuals, the total prevalence of PD ≥ 4 mm and ≥ 6 mm was 32.3% and 6.0%, respectively (Table 3 and Table S3). Among dentate individuals, the average mean PD was 1.89 (95% CI: 1.87–1.91). Women had a lower mean PD (1.83 mm; 1.81–1.86) than men (1.95 mm; 1.92–1.98), with an age‐related increase in mean PD observed. On average, 5.7% of sites, 1.0 teeth and 9.5% of teeth were affected by PDs of ≥ 4 mm. Sex and age‐related differences, as seen for mean PD, were consistent for all PD variables.

**TABLE 3:** Overview on prevalence, severity and extent of probing depths using Level 2 data from NAKO. Data are presented in total and stratified by sex, age and study region.

	*N*_total_ (unweighted)	*N*_dentates_ (unweighted)	Mean PD, mm	PD ≥ 4 mm				
				Prevalence, %^a^	Prevalence, %	Percentage of affected sites, %	Number of affected teeth	Percentage of affected teeth, %
Total	18,389	18,216	1.89 (1.87–1.91)	32.3 (31.0–33.6)	32.8 (31.5–34.1)	5.7 (5.3–6.1)	1.0 (1.0–1.1)	9.5 (9.0–10.1)
By sex								
Female	8756	8672	1.83 (1.81–1.86)	30.0 (28.3–31.8)	30.0 (28.3–31.8)	4.9 (4.5–5.4)	0.9 (0.8–1.0)	8.3 (7.6–9.1)
Male	9633	9544	1.95 (1.92–1.98)	35.7 (33.8–37.7)	35.7 (33.8–37.7)	6.5 (5.9–7.0)	1.2 (1.1–1.3)	10.7 (9.9–11.6)
By age								
19–29 years	1696	1696	1.63 (1.58–1.67)	14.7 (12.2–17.5)	14.7 (12.2–17.5)	1.8 (1.4–2.3)	0.4 (0.3–0.5)	3.1 (2.4–3.9)
30–39 years	1946	1946	1.78 (1.74–1.83)	26.4 (23.2–29.9)	26.4 (23.2–29.9)	4.0 (3.1–4.8)	0.9 (0.7–1.1)	6.9 (5.3–8.2)
40–49 years	4870	4867	1.92 (1.88–1.97)	34.9 (32.1–37.8)	34.9 (32.1–37.8)	6.0 (5.1–6.9)	1.2 (1.0–1.4)	10.0 (8.6–11.4)
50–59 years	5257	5220	2.00 (1.96–2.04)	39.5 (37.1–42.0)	40.1 (37.6–42.6)	7.5 (6.7–8.3)	1.2 (1.1–1.3)	12.2 (10.9–13.4)
60–74 years	4620	4487	2.08 (2.04–2.12)	42.2 (39.5–44.9)	44.8 (42.1–47.6)	8.5 (7.7–9.2)	1.3 (1.2–1.5)	14.3 (13.1–15.5)
By study region								
Augsburg	4362	4321	1.64 (1.61–1.68)	15.5 (13.2–18.1)	15.7 (13.4–18.3)	2.2 (1.7–2.6)	0.4 (0.3–0.5)	3.7 (2.9–4.5)
Mannheim	2643	2620	1.71 (1.65–1.76)	18.0 (15.5–20.9)	18.3 (15.7–21.2)	3.8 (2.8–4.8)	0.5 (0.4–0.7)	5.0 (3.8–6.2)
Muenster	1271	1263	1.69 (1.64–1.74)	12.8 (10.4–15.7)	12.8 (10.4–15.7)	1.6 (1.1–2.0)	0.3 (0.2–0.4)	2.4 (1.7–3.1)
Berlin‐Nord	1296	1282	2.05 (1.95–2.16)	51.0 (44.6–57.4)	52.6 (46.1–59.0)	8.6 (6.6–10.6)	1.7 (1.4–2.1)	15.8 (12.3–19.6)
Hannover	1409	1400	1.87 (1.80–1.95)	36.6 (32.0–41.6)	37.2 (32.5–42.1)	6.2 (4.9–7.4)	1.3 (1.0–1.6)	11.0 (8.8–13.2)
Hamburg	1553	1535	1.92 (1.86–1.97)	28.5 (24.3–33.2)	29.2 (24.8–33.9)	3.6 (2.7–4.6)	0.7 (0.5–0.9)	6.5 (4.7–8.4)
Kiel	1198	1188	1.65 (1.56–1.75)	29.3 (24.9–34.2)	29.6 (25.1–34.6)	6.8 (5.1–8.4)	1.1 (0.9–1.3)	9.1 (7.1–11.1)
Neubrandenburg	4657	4607	2.31 (2.28–2.34)	57.1 (54.7–59.4)	58.0 (55.7–60.4)	10.7 (9.9–11.5)	2.0 (1.8–2.1)	18.5 (17.3–19.7)

*Note:* Data are presented as survey‐weighted estimates with 95% confidence intervals.

^a^ Including edentulous individuals in the denominator (edentulous individuals were counted as non‐prevalent individuals; information from Level 1 tooth counts was considered to define edentulism).

Clear regional differences were observed in the PD variables (Table 3 and Table S3, Figure 2). The lowest prevalence of PD ≥ 4 mm was observed in Muenster (12.8%), Augsburg (15.5%) and Mannheim (18.0%) in the south and south‐west. The highest prevalence was observed in Berlin‐Nord (51.0%) and Neubrandenburg (57.1%), both located in the north‐east. Similar regional patterns were observed for the prevalence of PD ≥ 6 mm and mean PD, for which disparities were clinically relevant (i.e., > 0.5 mm for at least one pairwise comparison; range 1.64–2.31 mm). Regional differences were also apparent across all age groups (Figure S2).

**FIGURE 2:**
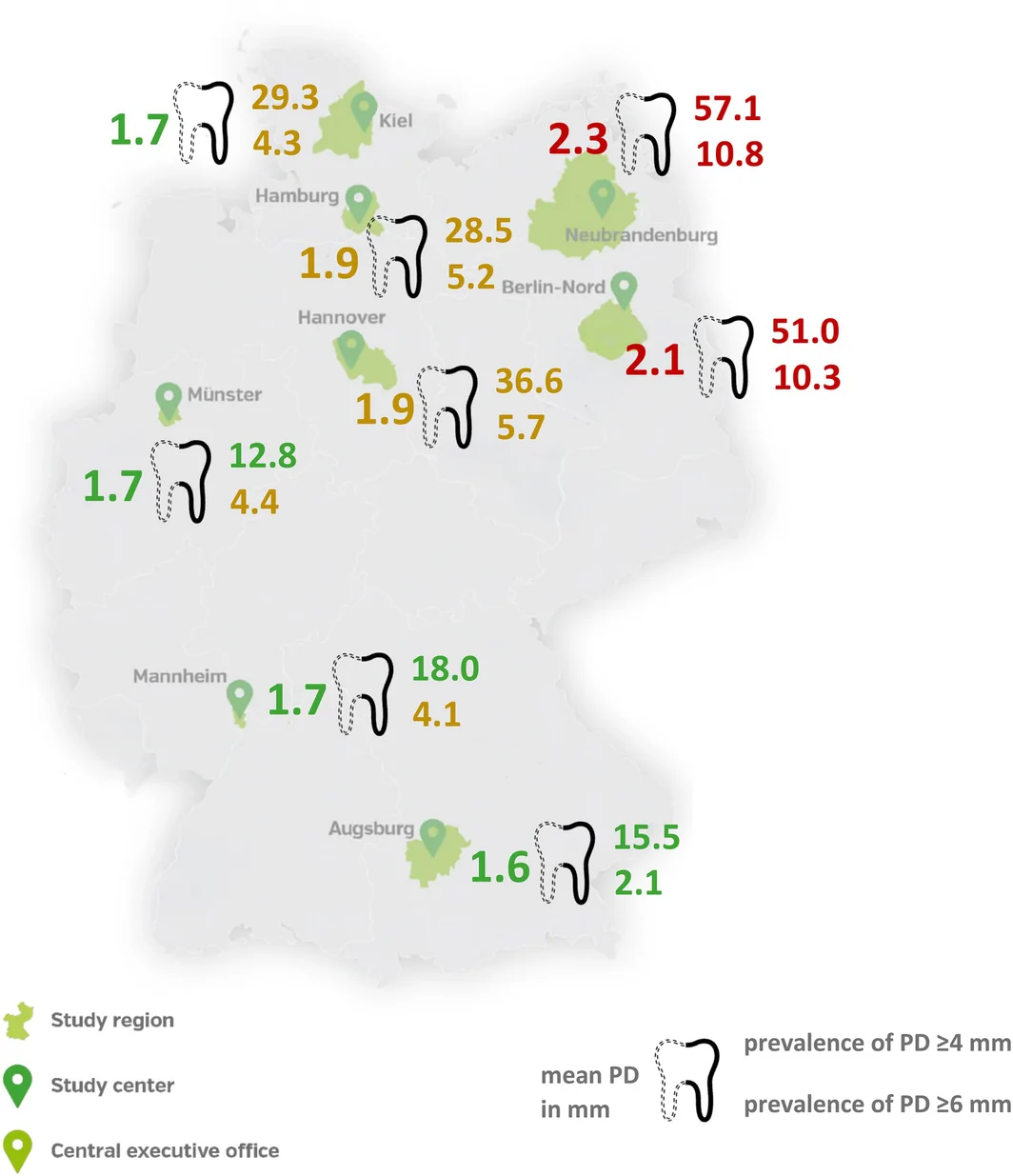
Coloured map showing mean probing depth (left) and prevalences of probing depths ≥ 4 mm and ≥ 6 mm (right; including edentulous individuals in the denominator) for each of the Level 2 study regions of NAKO.

According to regression models (Table 4), prevalence of PD ≥ 4 mm and mean PD were lower in all study regions other than Neubrandenburg, with disparities being most pronounced for Augsburg, Mannheim and Muenster. Even after adjustment for education, smoking status and diabetes (model 2), effect estimates remained stable.

**TABLE 4:** Regression models evaluating associations between study region and the prevalence of probing depths ≥ 4 mm and mean probing depth (*N* = 16,269; Level 2).

	Prevalence of PD ≥ 4 mm		Mean PD	
	Model 1	Model 2	Model 1	Model 2
Using complete case data				
Study region (ref. Neubrandenburg)	IRR (95% CI)	IRR (95% CI)	MR (95% CI)	MR (95% CI)
Augsburg	0.29 (0.27–0.31)	0.29 (0.27–0.31)	0.72 (0.71–0.73)	0.71 (0.71–0.72)
Mannheim	0.34 (0.32–0.37)	0.35 (0.32–0.38)	0.74 (0.73–0.76)	0.75 (0.73–0.76)
Muenster	0.29 (0.25–0.33)	0.30 (0.26–0.34)	0.75 (0.74–0.77)	0.76 (0.75–0.78)
Berlin‐Nord	0.94 (0.89–1.003)	0.97 (0.91–1.03)	0.91 (0.89–0.93)	0.91 (0.89–0.93)
Hannover	0.67 (0.62–0.72)	0.68 (0.63–0.74)	0.81 (0.80–0.83)	0.82 (0.80–0.83)
Hamburg	0.53 (0.49–0.57)	0.53 (0.49–0.57)	0.86 (0.84–0.87)	0.86 (0.84–0.88)
Kiel	0.57 (0.52–0.62)	0.56 (0.52–0.62)	0.75 (0.73–0.77)	0.75 (0.73–0.77)
Using multiply imputed data				
Study region (ref. Neubrandenburg)				
Augsburg	0.29 (0.27–0.32)	0.29 (0.27–0.31)	0.72 (0.71–0.73)	0.72 (0.71–0.73)
Mannheim	0.35 (0.32–0.37)	0.35 (0.32–0.38)	0.74 (0.73–0.76)	0.75 (0.74–0.76)
Muenster	0.29 (0.25–0.33)	0.29 (0.26–0.33)	0.76 (0.75–0.78)	0.77 (0.75–0.78)
Berlin‐Nord	0.96 (0.91–1.02)	0.99 (0.93–1.04)	0.91 (0.89–0.93)	0.92 (0.90–0.94)
Hann‐over	0.67 (0.62–0.72)	0.68 (0.63–0.73)	0.81 (0.79–0.83)	0.82 (0.80–0.83)
Hamburg	0.53 (0.49–0.58)	0.53 (0.49–0.58)	0.86 (0.85–0.88)	0.86 (0.85–0.88)
Kiel	0.55 (0.51–0.60)	0.55 (0.51–0.60)	0.74 (0.72–0.76)	0.74 (0.72–0.76)

*Note:* Modified Poisson (for prevalence of PD ≥ 4 mm; with robust standard errors; reports incidence rate ratios [IRR] with 95% confidence intervals [CI]) and generalised linear regression models (for mean PD; with a gamma distribution and log link; reports mean ratios (MR) with 95% confidence intervals). Model 1 was adjusted for age (10‐year groups) and sex; model 2 was additionally adjusted for theoretical education, smoking status and diabetes mellitus.

Abbreviation: PD, probing depth.

### 3.4 Dental Interview Data (Level 1)

The prevalences of a dentist's diagnosis of periodontitis, loose teeth and bleeding gums when brushing teeth were 35.1%, 7.1% and 20.5%, respectively (Table 5). With increasing age, the prevalences of a dentist's diagnosis of periodontitis and loose teeth increased steadily, while the prevalence of bleeding gums decreased. Regional patterns differed across the three items.

**TABLE 5:** Results from self‐reported interview data using Level 1 data from NAKO. Data are presented in total and stratified by sex, age and study region.

	Unweighted *N*	Prevalence of dentist's diagnosis of periodontitis, %	Unweighted *N*	Prevalence of having loose teeth, %	Unweighted *N*	Prevalence of bleeding gums when brushing teeth, %
Total	159,370	35.1 (34.7–35.5)	185,008	7.1 (6.9–7.4)	188,663	20.5 (20.2–20.9)
By sex						
Female	82,151	34.6 (34.0–35.1)	92,522	6.7 (6.4–7.0)	94,487	21.0 (20.5–21.5)
Male	77,219	35.7 (35.1–36.3)	92,486	7.6 (7.3–8.0)	94,176	20.0 (19.5–20.5)
By age						
19–29 years	16,286	8.7 (8.0–9.3)	18,927	1.9 (1.6–2.3)	19,288	22.6 (21.7–23.6)
30–39 years	16,736	23.3 (22.3–24.3)	20,288	3.7 (3.2–4.2)	20,656	22.8 (21.9–23.8)
40–49 years	41,471	36.9 (36.1–37.8)	49,292	7.1 (6.6–7.6)	50,131	21.0 (20.4–21.7)
50–59 years	43,178	49.1 (48.3–50.0)	49,700	11.0 (10.4–11.5)	50,698	19.9 (19.3–20.6)
60–74 years	41,699	54.3 (53.5–55.1)	46,801	11.5 (11.0–12.0)	47,890	15.9 (15.3–16.5)
By study region						
Augsburg/Regensburg	24,014	34.6 (33.3–35.9)	28,366	7.3 (6.6–8.1)	28,898	19.9 (18.9–21.0)
Mannheim/Freiburg/Saarbruecken	24,299	32.4 (31.3–33.5)	28,019	7.0 (6.3–7.6)	28,443	21.2 (20.3–22.2)
Essen/Muenster/Duesseldorf	23,693	35.9 (34.9–36.8)	26,648	6.5 (6.0–7.0)	27,260	19.4 (18.6–20.2)
Halle/Leipzig	16,531	34.7 (33.6–35.8)	19,274	7.2 (6.6–7.8)	19,756	22.0 (21.9–23.0)
Berlin‐Süd/Mitte/Nord	24,512	36.1 (35.1–37.0)	28,161	7.5 (7.0–8.0)	28,792	20.9 (20.1–21.7)
Hannover/Hamburg/Bremen	23,868	35.8 (34.8–36.8)	27,293	7.4 (6.9–7.9)	27,998	20.7 (19.8–21.6)
Kiel	7678	34.4 (32.5–36.3)	8712	6.9 (5.9–7.8)	8904	16.5 (15.1–17.9)
Neubrandenburg	14,775	37.3 (36.1–38.5)	18,535	7.1 (6.5–7.8)	18,612	21.6 (20.6–22.5)

*Note:* Data are presented as survey‐weighted prevalences with 95% confidence intervals. *N*, number.

### 3.5 Comparative Prevalence of Edentulism and Tooth Counts in 65–74‐Year‐Olds

We conducted the following comparisons in individuals aged 65–74 (Tables S4 and S5 for study participant characteristics): Firstly, we compared regional tooth count patterns from NAKO with those from the DMS V study. Secondly, we compared tooth counts from Neubrandenburg with those from SHIP‐TREND‐0. In NAKO, the prevalence of edentulism ranged from 4.9% in Augsburg/Regensburg to 7.5% in Essen/Muenster/Düsseldorf. However, disparities across study regions were clinically irrelevant (< 5%). Among dentate individuals, the mean number of teeth ranged from 18.6 in Neubrandenburg to 21.5 in Berlin. More pronounced differences in terms of edentulism and tooth count were found in DMS V. The best results were found in the north‐west and south (both with edentulism at 5.5% and an average of 19.9 and 19.7 teeth in dentates, respectively). The worst results were found in the mid‐west (edentulism at 19.2%; 20.0 teeth in dentates). Secondly, compared hto Neubrandenburg (edentulism at 6.0%; 18.6 teeth in dentates), notably worse values were found in SHIP‐TREND‐0 (edentulism at 15.7%; 15.7 teeth in dentates).

## 4 Discussion

NAKO is the first population‐based cohort study to evaluate regional differences in the prevalence of edentulism, the number of teeth as well as the prevalence, severity and extent of PDs in Germany. The total prevalence of edentulism was 1.7%, with an average of 25.8 teeth among dentate individuals. The total prevalence of PD ≥ 4 mm and ≥ 6 mm (including edentulous individuals in the calculation) was 32.3% and 6.0%, respectively, with an average mean PD of 1.89 mm in dentates. Consistent sex‐ and age‐related disparities were evident across all indicators of tooth loss and PD. Finally, regional disparities were consistently evident, with higher prevalences of edentulism and PDs in the north‐east and the lowest in the south‐west.

When supplementing data from other German cohorts, we must acknowledge that different examiner qualifications and recording protocols made comparison of PDs across studies difficult (Holtfreter et al. 2015). Therefore, we restricted our comparisons to tooth loss data in individuals aged 65–74, as this data can be collected more robustly. While there were consistent regional disparities in the prevalence of edentulism and the number of teeth across the three studies, the estimates varied considerably. For instance, the prevalence of edentulism was 6.0% in the Neubrandenburg study region, 13.4% in north‐east/east Germany (DMS V) and 15.7% in SHIP‐TREND‐0. While differences between NAKO (2014–2019), DMS V (2013–2014) and SHIP‐TREND‐0 (2008–2012) can be explained by well‐documented cohort effects (Schutzhold et al. 2015), comparing prevalence across studies is difficult because of differences in response rates, rurality of the study regions, examiner qualifications and sample sizes. Furthermore, the proportion of missing teeth attributable to periodontitis versus other causes is unknown and is likely to vary by age group and study region. In Germany, tooth extractions due to caries, for orthodontic reasons or wisdom tooth removal are quite common among subjects under 40 (Glockmann et al. 2011). However, after the age of 30, periodontitis emerges as the main cause of tooth extraction, becoming the primary reason by the age of 40. Thus, for participants aged 65–74, tooth counts probably reflect a lifetime's experience of caries and periodontitis experience, which limits the interpretability of the findings.

NAKO was the first nationwide, population‐based cohort to systematically assess regional disparities in tooth loss and PDs across Germany. However, although regional disparities were statistically evident, they were clinically relevant only for the number of teeth in dentates (≥ 1), the prevalence of PD ≥ 4 mm (> 5%), the prevalence of PD ≥ 6 mm (> 5%) and mean PD (> 0.5 mm). While the lowest average tooth count was observed in Neubrandenburg (24.1), disparities between the other study regions were less pronounced, that is, on average < 0.6 teeth. The prevalence of PD ≥ 4 mm or ≥ 6 mm ranged from approximately 15% and 3%, respectively, in the study regions of Augsburg, Mannheim and Muenster to 55% and 10%, respectively, in BerlinNord and Neubrandenburg. Similar studies have been conducted in the United States using the National Health and Nutrition Examination Survey database (Eke et al. 2016). Here, regional patterns of periodontitis coincided with those of known periodontal risk factors. The highest prevalence was found in areas with a higher proportion of older adults of lower socio‐economic status, large immigrant populations, a high density of tobacco users and limited access to dental care.

In NAKO, regional differences in periodontitis prevalence may be explained by regional differences in the major periodontal risk factors, such as smoking (Vogt et al. 2017) and diabetes mellitus (Schipf et al. 2014). However, while region‐specific risk factor distributions partly aligned with periodontitis prevalences, regression models indicated that disparities among study regions only marginally reflected compositional differences in these risk factors, as regional differences did not decrease after adjustment for the same. Thus, the observed disparities in periodontitis prevalence and severity across the NAKO study regions may be more related to variations in socio‐economic structures (Schuch et al. 2017), financial resources, degree of urbanisation, accessibility of professional dental care and shopping facilities (dental hygiene products), iatrogenic factors and the long‐term consequences of treatment policies.

Socio‐economic structures still vary greatly (Schwarze 2023), with significant disparities in education and prosperity between study regions, with the lowest levels being found in rural areas. These disparities are mainly the result of the historical development of the political and healthcare systems in the former German Democratic Republic and Federal Republic of Germany. In line with the idea that socio‐economic status is widely considered as the ‘cause of the causes’ of health inequalities (Marmot and Allen 2014), disparities in socio‐economic status might explain a major portion of the observed differences. Similar results were reported in an American study (Warren et al. 2025), which demonstrated that educational attainment greatly impacts the risk of developing periodontitis in midlife. Potential pathways include improved oral hygiene behaviour, including regular interdental cleaning (Holtfreter et al. 2024), an increased use of electric toothbrushes (Sager et al. 2023), a greater likelihood of preventive dental visits for screening purposes (Chugh et al. 2026) and a higher purchasing power (Psacharopoulos and Patrinos 2018).

Region‐specific disparities in the prevalence of PDs may also be explained by disparities in periodontal treatment and care. This study found that the prevalence of self‐reported dentist's diagnoses of periodontitis showed small variations, ranging from 32.4% in the Mannheim cluster to 37.3% in Neubrandenburg (Table 5). However, it should be noted that reporting bias is likely, so this information is not very reliable. An analysis by the statutory health insurance provider BARMER revealed that the utilisation rate of diagnostic and therapeutic services was highest in Bavaria (29.3%; covering Augsburg) and lowest in Lower Saxony (20.7%; covering Muenster and Hannover) (Rädel et al. 2021). Thus, lower prevalences of PD ≥ 4 mm found in the study regions Augsburg, Muenster and Hannover might partly be explained by these differences. It is also noteworthy that in all federal states, the figures for diagnostic services (27.0% in total) were significantly higher than those for therapeutic services (1.9% in total). Furthermore, the long‐term reduction in PDs depends, among other things, on the quality of treatment (university vs. general practice vs. specialist) and successful long‐term involvement of the patient in long‐term periodontal care (Kocher, Lösler, et al. 2025). Using registry data, it was shown that periodontal treatment did not lead to improved tooth retention (Kocher et al. 2022) and therefore cannot be considered effective. Taken together, the use of periodontal therapy explains only a small portion of the differences between the study regions.

Also, temporal changes towards a higher prevalence of highly educated individuals accounted for a significant proportion of the observed change in the number of teeth in three repeated cross‐sectional studies from Germany and Sweden (Pitchika et al. 2022). Among iatrogenic risk factors, insufficient or protruding filling margins or crown margins combined with a poorer ability of dental cleaning and biofilm removal might trigger periodontal inflammation and progression of the disease (Nafz et al. 2026).

This study has strengths and limitations. A notable strength is the study's large‐scale, population‐based design. Another major advantage is the identical assessment of dental and periodontal health in all 18 NAKO study centres, which enables a valid comparison across study sites and an assessment of regional variations in PDs and tooth counts across Germany. High‐quality standards for oral examinations in NAKO are ensured through the comprehensive theoretical and practical training of all study nurses, which was conducted by experienced dentists (Samietz et al. 2025). Additionally, a reliability study was performed, which indicated that the collected data were reliable (Samietz et al. 2025). Finally, comprehensive data from three large‐scale, population‐based cohort studies were additionally available for detailed analysis of regional disparities.

Some limitations should be noted. Firstly, the response rate of 15.6% was low. However, it was within the range reported by other recently conducted large‐scale population‐based cohort studies (Fry et al. 2017; Völzke et al. 2022). Interestingly, response rates varied across the NAKO study regions (Rach et al. 2025), with the highest response proportions being found in rural areas. Although calibration weights compensate for differential non‐response with respect to measured characteristics (e.g., age, sex, nationality, education level, migration background and household size), they do not directly correct for unmeasured factors associated with participation and oral health, such as health consciousness. To quantify the extent to which the bias of healthy volunteers may differ across regions, we evaluated the correlation between general practitioner visit patterns and responses. Indeed, there was a strong correlation between general practitioner visit patterns and response (*r*_P_ = 0.70; Figure S3), with higher response rates observed in regions with a high proportion of participants who reported at least one visit to a general practitioner in the last 12 months. Therefore, if we assume that responders are more health conscious than non‐responders, the mean PD estimates within study regions are likely to have been underestimated. Therefore, the ‘true’ estimates of mean PD would be much higher in study regions with the lowest response rates (e.g., Berlin‐Nord, Hannover, Mannheim and Hamburg), moderately higher in study regions with modest response rates (e.g., Kiel, Muenster and Neubrandenburg) and slightly higher in study regions with the highest response (e.g., Augsburg). Thus, the ‘true’ differences between the most extreme study regions (i.e., Augsburg and Neubrandenburg) would probably be even greater. Secondly, as we did not measure clinical attachment levels (CALs), the findings cannot be translated into the 2018 classification of periodontitis. A discussion of further limitations can be found in the Supporting Information.

## 5 Conclusion

Periodontitis remains prevalent, representing a significant public health concern in Germany. Findings of regional patterns highlight potential areas for region‐specific preventive and treatment approaches. Demographic trends, an increased number of periodontally exposed teeth and rising trends in known periodontal risk factors such as Type 2 diabetes and poor nutrition suggest that demand for treatment will increase in the future. The results contribute to understanding and addressing current public health priorities, but the cross‐sectional nature of the data does not allow direct assessment of disease progression or future treatment needs. Future research could focus on evaluating the impact of risk factors such as diabetes, nutrition and oral hygiene and care to guide preventive strategies, adequate treatment and care planning and allocation of financial resources.

## Author Contributions

J.G. and B.H. drafted the manuscript. J.G., T.K., P.K., A.W., S.R. and B.H. contributed to the conception or design of the work. B.H., D.S., K.K., K.B. and S.Ra. contributed to the analysis. G.A., K.H.G., D.H., T.‐S.K., J.K., T.P., A.A., A.C., Y.K., L.K., M.L., C.M.‐F., R.M., N.O., R.P., N.P., W.R., M.B.S., Sa.S., B.S., S.N.W., S.Ra., S.R., A.R.J., H.V. and T.K. contributed to the design and acquisition of the data. All authors contributed to the interpretation of data, critically revised the manuscript and approved the final version. All authors agreed to be accountable for all aspects of the work in ensuring that questions related to the accuracy or integrity of any part of the work are appropriately investigated and resolved.

## Funding

NAKO: This project was conducted using data (Application No. NAKO‐550) from the German National Cohort (NAKO) (www.nako.de). The NAKO is funded by the Federal Ministry of Research, Technology and Space (BMFTR) [project funding reference numbers: 01ER1301A/B/C, 01ER1511D and 01ER1801A/B/C/D and 01ER2301A/B/C], the federal states of Germany and the Helmholtz Association, the participating universities and the institutes of the Leibniz Association.

SHIP‐TREND‐0: SHIP‐TREND‐0 is part of the Community Medicine Research Network of the University Medicine Greifswald, which is supported by the German Federal State of Mecklenburg‐West Pomerania.

DMS V: The German Oral Health Studies are financed by the German dental profession via the German Dental Association (Bundeszahnärztekammer [BZÄK]) and the National Association of Statutory Health Insurance Dentists (Kassenzahnärztliche Bundesvereinigung [KZBV]).

## Ethics Statement

NAKO: All procedures performed involving human participants were in accordance with the ethical standards of the institutional and/or national research committee and are in accordance with national law and with the Declaration of Helsinki of 1975 (in the current, revised version). The research protocol was approved by the Ethics Committee of the Medical Faculty of the Ludwig Maximilians‐University of Munich/Bayerische Landesärztekammer; Nr. 13023 and all other local Ethics Committees of the study centres. Written informed consent was obtained from all individual participants included in the study. Trial registration German National Cohort (NAKO): DRKS00037328.

DMS V: This research was conducted in accordance with recognised ethical standards for research practice as outlined in the Declaration of Helsinki. The study protocol of the DMS V was reviewed and approved by the Institutional Review Board (IRB) of the North Rhine State Chamber of Physicians, Duesseldorf (DMS V registration number 2013384). This study is registered at the German Health Services Research Data Bank (DMS V registration number VfD_DMSV_13_002152). Each participant received detailed information about the study and provided written consent before enrolment.

SHIP‐TREND‐0: Written informed consent was obtained from all participants in accordance with the principles of the Declaration of Helsinki. SHIP‐TREND‐0 was positively evaluated by the ethics committee of the University of Greifswald (BB 39/08a). All participants were informed about the study protocol and signed the informed consent and the privacy statement.

## Conflicts of Interest

The authors declare no conflicts of interest.

## Supporting information


**Table S1:** Characteristics of Level 1 participants of NAKO (2014–2019) stratified by study region (*N* = 184,871).
**Table S2:** Characteristics of Level 2 participants of NAKO (2014–2019) stratified by study region (*N* = 18,389).
**Table S3:** Overview on prevalence, severity and extent of probing depths ≥ 6 mm using Level 2 data from NAKO. Data are presented in total and stratified by sex, age and study region.
**Table S4:** Data on edentulism and number of teeth (including third molars) in participants aged 65–74 in the German National Cohort (NAKO; Level 1), the Fifth German Oral Health Study (DMS V) and the Study of Health in Pomerania (SHIP‐TREND‐0).
**Table S5:** Characteristics of participants aged 65–74 in the German National Cohort (NAKO; Level 1), the Fifth German Oral Health Study (DMS V) and the Study of Health in Pomerania (SHIP‐TREND‐0).
**Figure S1:** Distribution of Level 1 and Level 2 study centres within Germany.
**Figure S2:** (A) Number of missing teeth (including edentulous persons) according to age within Level 1 study regions and (B) mean probing depth according to age within Level 2 study regions of NAKO.
**Figure S3:** Correlation between and response for Level 1 study regions.

## Data Availability

NAKO: NAKO data are generally not available to the public due to strict data protection regulations. However, scientists can apply for data use according to the official usage regulation specifications. Please refer to https://transfer.nako.de for further information. DMS V: DMS V data are generally not available to the public due to strict data protection regulations. SHIP‐TREND‐0: Restrictions apply to the availability of SHIP‐TREND‐0 data, which were used under licence for this study. Data are available from https://transfer.ship‐med.uni‐greifswald.de/FAIRequest/data‐use‐explore with the permission of Forschungsverbund Community Medicine.
