# Supplementary material for: Regional Disparities in the Prevalence of Probing Depths and Tooth Loss in Germany—Results From the German National Cohort (NAKO)
Source: J Clin Periodontol. 2026 Sep 18;53(12):1785–97. doi: 10.1111/jcpe.70201 (PMC13751319; doi:10.1111/jcpe.70201)
Supplement: Supplementary file 1 [file JCPE-53-1785-s001.docx]

# Supplemental Material

# Regional Disparities in the Prevalence of Probing Depths and Tooth Loss in Germany – Results from the German National Cohort (NAKO)

Jana Gortschakow, Katrin Borof, Dominic Susanna, Kathrin Kuhr, Ghazal Aarabi, Sebastian-Edgar Baumeister, Antonio Ciardo, Karin Halina Greiser, Daniel Hagenfeld, Katrin Hertrampf, Ti-Sun Kim, Jan Kühnisch, Tobias Pischon, Stefan L. Reckelkamm, Stefanie Samietz, Martin Wojciechowsky, Asma’a Alselwi, Yvonne Kemmling, Lilian Krist, Michael Leitzmann, Claudia Meinke-Franze, Rafael Mikolajczyk, Nadia Obi, Regina Pickford, Nicole Pischon, Wolfgang Rathmann, Matthias B. Schulze, Sabine Schipf, Börge Schmidt, Stefan N. Willich, Stefan Rach, A. Rainer Jordan, Henry Völzke, Thomas Kocher, Philipp Kanzow, Alexander Welk, Stefan Rupf, Birte Holtfreter

# Material and Methods

## NAKO

#### Study design

Two levels of examination intensity were employed. The standard Level 1 programme was offered to all participants at all 18 study centres. In addition to interviews, questionnaires, and comprehensive medical assessments, tooth counts (including third molars) were also recorded. Figure 1 of Samietz et al. (2025) provides a detailed flowchart for Level 1 dental interview and oral examinations. Additionally, in-depth examinations were offered to 20% randomly selected participants (Level 2). Each study centre selected one of three modules to be completed by all Level 2 participants, one of which was the comprehensive oral examination. As a result, Level 2 oral examinations were performed in eight centres. In the remaining study centres, Level 2 oral examinations were performed on a smaller number of Level 2 participants (approximately 100 per study centre); these data were excluded from the analysis. The Level 2 oral examinations included assessments of the dental and periodontal status. Figure 2 of Samietz et al. (2025) provides detailed information on sample size reductions for dental and periodontal examinations. For this study, data from Level 1 and Level 2 examinations were combined for analysis, yielding a total of 18,389 individuals.

#### Level 1 examinations

Level 1 examinations included the tooth count (including third molars) and a dental interview. The latter included questions about i) previous dentist’s diagnosis of periodontitis or periodontitis (lifetime); ii) having loose teeth; and iii) bleeding gums when brushing teeth.

#### Level 2 dental and periodontal examination

#### Level 2 dental examinations included a tooth status and PDs (Samietz et al., 2025). The number of present teeth (excluding third molars, half mouth) was calculated. PDs were recorded at two sites (mesiobuccal and midbuccal) per tooth using a PCP-UNC 15 manual probe (0.25 N pressure). The half-mouth protocol was applied, with third molars excluded. The side (left or right) was chosen at random. However, due to a software error, more right sides were examined at the beginning of the study, resulting in 62.7% of examinations being of the right side (varying between 55.8% in Mannheim and 70.1% in Augsburg) and 37.3% of examinations being of the left side. PD was defined as the distance between the gingival margin and the pocket base. Measurements were rounded down to the nearest whole millimetre. Participants who answered ‘yes’ or ‘don’t know’ to the question about having a heart pass were excluded from the periodontal examination. In total, the proportion of participants with a heart pass was very low (1.1%). Across study regions, this proportion ranged between 0.3% in Berlin North and 1.4% in Muenster.

#### Reliability data

All dental examinations were conducted by study nurses who underwent training and calibration by experts to ensure reliability and reproducibility, following a standardised protocol (Samietz et al., 2025). A reliability study was performed, in which NAKO participants were re-examined after an average of 270 days (N=794; standard deviation (SD) 82.7; range 35-434) and 274 days (N=359; SD 80.5; range 49-434) for Level 1 and Level 2 examinations, respectively. The intra-class correlation (ICC) coefficient for Level 1 number of teeth (including third molars) was 0.94 (95% CI: 0.93-0.95). For Level 2 dental status assessments, intra-class kappa was 0.88 (SE 0.01) and agreement was 97.4%. For Level 2 PDs (site level) the ICC coefficient was 0.74 (95% CI: 0.73-0.75).

#### Covariates

Age was recorded in years at the time of the baseline examination. Education was recorded according to the International Standard Classification of Education (ISCED-97) and categorised as ≤10 years, 11–13 years, 14–17 years, and ≥18 years based on the number of theoretical education years completed by the participant (UNESCO, 1999; Dragano et al., 2020). Smoking status was assessed by self-report and categorised as never, former, or current smoking. Diabetes mellitus was defined as self-reported lifetime physician’s diagnosis. The number of dental visits within the last 12 months was recorded and categorised as ‘Having a dental visit within the last 12 months’ (yes/no).

## DMS V

#### Study design

The Fifth German Oral Health Study (DMS V) is a stratified, multi-stage, cross-sectional, nationwide study of the German population with examinations being conducted between 2013 and 2014 (Jordan et al., 2014). Study participants were drawn from local residents’ registration offices in 90 randomly selected communities (sample points) using a cluster-random sampling stratified for regions and areas of urbanisation. A disproportional sample point selection was performed with 60 study sample points in the western federal states of Germany and 30 study sample points in the eastern states. Examinations were conducted between October 2013 and July 2014, including 4609 individuals (response rate 50.1%). For the analysis, the sample included 1,042 individuals aged 65 to 74.

Detailed information about the sampling design, data collection protocols, and data availability can be found elsewhere (Jordan et al., 2014). In this study, we report tooth loss data from participants aged 65-74. Five regions were defined for data presentation, which roughly correspond to the NAKO regions: north-east/east (Mecklenburg-Western Pomerania/ Brandenburg/ Berlin/ Saxony-Anhalt/ Saxony/ Thuringia), north-west (Schleswig-Holstein/ Hamburg/ Bremen/ Lower Saxony), mid-west (North Rhine-Westphalia), south-west (Baden-Württemberg/ Saarland/ Hesse/ Rhineland-Palatinate), and south (Bavaria).

#### Dental examination

#### Tooth-specific findings were obtained for all teeth including third molars. The following findings were recorded: extracted teeth due to caries, not replaced; missing, non-replaced teeth, missing for reasons other than caries; full crown; partial crown (at least one cusp covered); anchor crown (bridge anchor, telescopic crown, crown anchored to bar, root caps); pontic (fixed prosthesis); replaced tooth (removable prosthesis); implant (with prosthetic restoration). The number of present teeth was calculated including third molars.

#### Reliability data

To ensure high quality dental assessments, examiners were trained and calibrated by experts. Reliability tests were performed once before the field phase and twice during the field phase. To test interrater reliability, examiners were tested against experienced dentists. In addition, dental examinations were repeated by the examiners to assess intra-rater reliability. Cohen’s kappa reliability coefficients were 0.80 (intra-rater) and 0.76 (inter-rater) for dental status (Jordan et al., 2014).

#### Covariates

Age was recorded in years at the time of the baseline examination. Information on school education was categorised as less than 10 years, 10 years, more than 10 years, or another school-leaving qualification. Smoking status was classified as never, former, or current smoking. Self-reported physician’s diagnosis of diabetes mellitus was recorded.

#### Survey weighting

For DMS V, sampling weights were used to adjust for different probabilities of subject selection and differences in sex, age, region, and school education with respect to the base population (Jordan et al., 2014).

## SHIP-TREND-0

#### Study design

SHIP-TREND is a population-based cohort study conducted in Western Pomerania, a region in north-east Germany (Völzke et al., 2022). A stratified random sampling was employed to select 10,000 adults aged 20–79 from regional population registries, leveraging the centralised system in Mecklenburg-Western Pomerania. The sampling was stratified by age, sex, and city/county of residence. In total, 4,420 individuals participated in the baseline examinations (2008-2012; SHIP-TREND-0), achieving a response of 50.1%. In this study, we reported tooth loss data from 756 participants aged 65–74.

#### Dental examination

Dental examinations were conducted in a dental chair under operatory light, with the option to use aspiration or an air jet if required; magnification glasses were not permitted. The number of teeth present was calculated from dental status records, including root remnants and third molars.

#### Covariates

Age was recorded in years at the time of the baseline examination. School education was collected based on participants’ reported years of schooling and categorised as <10 years, 10 years, or >10 years. Smoking status was classified as never, former, or current smoking. Self-reported physician’s diagnosis of diabetes mellitus was recorded.

#### Reliability data

Dental examinations were conducted by six calibrated examiners. Five persons not connected to the study were repeatedly examined after seven days for reliability exercises. For assessment of the tooth status, Cohen’s kappa reliability coefficients were 0.93–0.99 (intra-examiner) and 0.94–0.98 (pairwise inter-examiner).

#### Survey weighting

Survey weights multiplicatively combined the design weights (post-stratification weights from the population size and the target sample) with non-response weights; in addition, the strata of the sampling process were considered.

# Discussion

#### Limitations

Secondly, although the study nurses received extensive training and calibration throughout the study, it should be noted that they had no prior knowledge of dentistry and we refrained from measuring clinical attachment levels (CAL) in all study waves. Consequently, international comparisons of CAL distributions and the 2018 classification of periodontitis could not be evaluated. Moreover, since PDs only reflect current inflammatory status, information on lifetime accumulation of periodontal damage is missing. Thirdly, Level 2 PDs were recorded at only two sites per tooth in a half-mouth manner, excluding the distobuccal and all lingual sites. It is widely acknowledged that such a protocol underestimates prevalence, severity, and extent estimates (Susin et al., 2005). However, it is conceivable that disparities in disease distribution may be attributable to region-specific risk factor patterns (e.g., smoking patterns affecting palatal sites). However, given the minimal disparities in smoking prevalence observed, it is plausible that underestimation may be uniform across the study regions. However, such data are still valuable to analyse regional disparities, assuming that the degree of underestimation does not differ across study centres. Fourthly, although the side of examination was supposed to be chosen at random, 62.7% of examinations were actually conducted on the right side due to a software error in the beginning pf the study period. This proportion varied from 55.8% in Mannheim to 70.1% in Augsburg. However, because of the bilateral symmetry of periodontitis (Mombelli et al., 2001; Darby et al., 2012), it can be assumed that the half-mouth recording sufficiently reflects the full-mouth recording. Fifthly, it was not possible to assess inter-rater agreement. Systematic differences in probing technique, probing force application and rounding behaviour between examiners and centres may have contributed to discrepancies in PD measurements and dental status recording. If these differences were centre-specific, they could explain a significant proportion of the observed regional variation, thereby generating apparent ‘regional disparities’ that are in fact measurement artefacts. To counteract such bias, the study nurses had received theoretical and practical training and certification. Reliability studies confirmed moderate intra-rater agreement (ICC=0.74; 95% CI: 0.73–0.75) for site-level PD measurements (Samietz et al., 2025). However, this still suggests that 26% of the variation in repeated PD measurements was due to measurement error. Thus, it cannot be excluded that regional differences in prevalences of PDs and tooth loss might in part be explained by inter-rater differences. Sixthly, 1.3% of Level 2 participants who had a heart pass or were unsure were excluded from the study. The exclusion of participants with a heart pass introduces a systematic selection bias, because individuals on anticoagulation are disproportionately older and more likely to have cardiovascular comorbidities, which are associated with periodontitis. However, given that disparities in proportions across study regions were negligible (ranging from 0.3% to 1.5%), differential selection bias can be considered negligible. Seventhly, the data from the three cohorts included were collected during slightly different time periods. Thus, secular improvements in preventive and restorative care over time may partly explain differences in PDs and tooth loss between studies. Finally, generalisability of prevalence estimates to the general German population is limited, as the weighting was not based on Germany’s overall population distribution, but on that of the regions actually covered by the NAKO study centres (Rach et al., 2025). Furthermore, since weights were calculated for each study centre individually, the weighting strategy does not target Germany as a whole, but rather the target populations of the individual study regions.

## References

Darby, I. B., Polster, A., Gan, J. S., Guo, Q., Henein, N., Heredia, A., . . . Radvar, M. (2012). Left-to-right distribution of periodontal disease. *Int J Dent Hyg, 10*(1), 74–79. doi:10.1111/j.1601-5037.2011.00509.x

Dragano, N., Reuter, M., Greiser, K. H., Becher, H., Zeeb, H., Mikolajczyk, R., . . . Schmidt, B. (2020). [Socio-demographic and employment-related factors in the German National Cohort (GNC; NAKO Gesundheitsstudie)]. *Bundesgesundheitsblatt Gesundheitsforschung Gesundheitsschutz, 63*(3), 267–278. doi:10.1007/s00103-020-03098-8

Jordan, R. A., Bodechtel, C., Hertrampf, K., Hoffmann, T., Kocher, T., Nitschke, I., . . . Micheelis, W. (2014). The Fifth German Oral Health Study (Fünfte Deutsche Mundgesundheitsstudie, DMS V) - rationale, design, and methods. *BMC Oral Health, 14*, 161. doi:10.1186/1472-6831-14-161

Mombelli, A., & Meier, C. (2001). On the symmetry of periodontal disease. *J Clin Periodontol, 28*(8), 741–745. doi:10.1034/j.1600-051x.2001.280804.x

Rach, S., Sand, M., Reineke, A., Becher, H., Greiser, K. H., Wolf, K., . . . Günther, K. (2025). The baseline examinations of the German National Cohort (NAKO): recruitment protocol, response, and weighting. *European Journal of Epidemiology, 40*(4), 475–489. doi:10.1007/s10654-025-01219-8

Samietz, S., Borof, K., Hertrampf, K., Aarabi, G., Ciardo, A., Finke, H., . . . Holtfreter, B. (2025). Dental and oral health assessments in the German National Cohort (NAKO). *BMC Oral Health, 25*(1), 149. doi:10.1186/s12903-025-05454-z

Susin, C., Kingman, A., & Albandar, J. M. (2005). Effect of partial recording protocols on estimates of prevalence of periodontal disease. *J Periodontol, 76*(2), 262–267. doi:10.1902/jop.2005.76.2.262

UNESCO. (1999). *Classifying Educational Programms - Manual for ISCED 97 Implementation in OECD Countries*. Retrieved from Paris: <http://www.oecd.org/education/1841854.pdf>

Völzke, H., Schössow, J., Schmidt, C. O., Jürgens, C., Richter, A., Werner, A., . . . Kocher, T. (2022). Cohort Profile Update: The Study of Health in Pomerania (SHIP). *International Journal of Epidemiology, 51*(6), e372–e383. doi:10.1093/ije/dyac034

Supplemental Table 1. Characteristics of Level 1 participants of the NAKO (2014-2019) stratified by study region (N=184,871).

|  | Augsburg/  Regensburg | Mannheim/  Freiburg/  Saarbruecken | Essen/  Muenster/  Duesseldorf | Halle/ Leipzig | Berlin  South/ Central/ North | Hanover/  Hamburg/  Bremen | Kiel | Neubrandenburg |
| --- | --- | --- | --- | --- | --- | --- | --- | --- |
| N | 27,791 | 27,088 | 28,189 | 17,138 | 29,592 | 28,612 | 8,861 | 17,600 |
| Age, years | 49.6±12.7 | 49.5±12.8 | 49.3±12.8 | 49.5±12.6 | 49.7±12.8 | 49.7±12.9 | 49.6±12.7 | 49.9±12.5 |
| Sex |  |  |  |  |  |  |  |  |
| Male | 14,079 (50.7%) | 13,402 (49.5%) | 13,958 (49.5%) | 8,530 (49.8%) | 14,540 (49.1%) | 14,023 (49.0%) | 4,353 (49.1%) | 8,958 (50.9%) |
| Female | 13,712 (49.3%) | 13,686 (50.5%) | 14,231 (50.5%) | 8,608 (50.2%) | 15,052 (50.9%) | 14,589 (51.0%) | 4,508 (50.9%) | 8,642 (49.1%) |
| Smoking status |  |  |  |  |  |  |  |  |
| Never | 13,634 (49.0%) | 12,700 (46.9%) | 12,581 (44.6%) | 7,813 (45.6%) | 12,650 (42.8%) | 12,150 (42.5%) | 3,820 (43.1%) | 7,395 (42.0%) |
| Former | 8,586 (30.9%) | 8,870 (32.8%) | 8,947 (31.7%) | 5,170 (30.2%) | 9,603 (32.4%) | 9,613 (33.6%) | 3,007 (33.9%) | 5,300 (30.1%) |
| Current | 5,165 (18.6%) | 4,521 (16.7%) | 5,107 (18.1%) | 3,875 (22.6%) | 6,003 (20.3%) | 5,329 (18.6%) | 1,700 (19.2%) | 4,048 (23.0%) |
| Missing | 406 (1.5%) | 997 (3.7%) | 1,554 (5.5%) | 280 (1.6%) | 1,336 (4.5%) | 1,520 (5.3%) | 334 (3.8%) | 857 (4.9%) |
| Years of theoretical education* |  |  |  |  |  |  |  |  |
| ≤10 years | 958 (3.4%) | 871 (3.2%) | 838 (3.0%) | 157 (0.9%) | 535 (1.8%) | 802 (2.8%) | 238 (2.7%) | 153 (0.9%) |
| 11-13 years | 8,693 (31.3%) | 6,324 (23.3%) | 6,391 (22.7%) | 4,431 (25.9%) | 5,816 (19.6%) | 7,645 (26.7%) | 2,409 (27.2%) | 5,982 (34.0%) |
| 14-17 years | 11,802 (42.5%) | 11,456 (42.3%) | 10,609 (37.6%) | 7,950 (46.4%) | 11,777 (39.8%) | 10,037 (35.1%) | 3,651 (41.2%) | 8,052 (45.7%) |
| ≥18 years | 4,056 (14.6%) | 6,129 (22.6%) | 7,381 (26.2%) | 3,263 (19.0%) | 8,365 (28.3%) | 7,850 (27.4%) | 1,704 (19.2%) | 1,794 (10.2%) |
| Missing | 2,282 (8.2%) | 2,308 (8.5%) | 2,970 (10.5%) | 1,337 (7.8%) | 3,099 (10.5%) | 2,278 (8.0%) | 859 (9.7%) | 1,619 (9.2%) |
| Known diabetes mellitus |  |  |  |  |  |  |  |  |
| No | 26,107 (93.9%) | 25,616 (94.6%) | 26,410 (93.7%) | 15,851 (92.5%) | 27,897 (94.3%) | 27,183 (95.0%) | 8,377 (94.5%) | 16,286 (92.5%) |
| Yes | 1,631 (5.9%) | 1,435 (5.3%) | 1,605 (5.7%) | 1,256 (7.3%) | 1,626 (5.5%) | 1,382 (4.8%) | 471 (5.3%) | 1,295 (7.4%) |
| Missing | 53 (0.2%) | 37 (0.1%) | 174 (5.6%) | 31 (0.2%) | 69 (0.2%) | 47 (0.2%) | 13 (0.2%) | 19 (0.1%) |
| Dental visit within the last 12 months |  |  |  |  |  |  |  |  |
| No | 2,955 (10.6%) | 2,831 (10.45%) | 3,105 (11.0%) | 1,446 (8.4%) | 2,831 (9.6%) | 2,809 (9.8%) | 824 (9.3%) | 1,291 (7.3%) |
| Yes | 17,183 (61.8%) | 16,914 (62.4%) | 17,701 (62.8%) | 11,976 (69.9%) | 19,185 (64.8%) | 18,289 (63.9%) | 5,862 (66.2%) | 9,992 (56.8%) |
| Missing | 7,653 (27.5%) | 7,343 (27.1%) | 7,383 (26.2%) | 3,716 (21.7%) | 7,576 (25.6%) | 7,514 (26.3%) | 2,175 (24.55%) | 6,317 (35.9%) |

Data are presented as mean ± standard deviation or N (%). * International Standard Classification of Education 97 (ISCED-97)

Supplemental Table 2. Characteristics of Level 2 participants of the NAKO (2014-2019) stratified by study region (N=18,389).

|  | Augsburg | Mannheim | Muenster | Berlin North | Hanover | Hamburg | Kiel | Neubrandenburg |
| --- | --- | --- | --- | --- | --- | --- | --- | --- |
| N | 4,352 | 2,643 | 1,271 | 1,296 | 1,409 | 1,553 | 1,198 | 4,657 |
| Age, years | 50.1±12.3 | 49.5±12.7 | 49.0±13.0 | 47.1±12.1 | 49.9±12.4 | 49.6±12.9 | 49.1±13.1 | 50.7±11.6 |
| Sex |  |  |  |  |  |  |  |  |
| Male | 2,318 (53.1%) | 1,441 (54.5%) | 667 (52.5%) | 726 (56.0%) | 712 (50.5%) | 755 (48.6%) | 569 (47.5%) | 2,445 (52.5%) |
| Female | 2,044 (46.9%) | 1,202 (45.5%) | 604 (47.5%) | 570 (44.0%) | 697 (49.5%) | 798 (51.4%) | 629 (52.5%) | 2,212 (47.5%) |
| Smoking status |  |  |  |  |  |  |  |  |
| Never | 2,052 (47.0%) | 1,257 (47.6%) | 636 (50.0%) | 668 (51.5%) | 647 (45.9%) | 683 (44.0%) | 543 (45.3%) | 2,174 (46.7%) |
| Former | 1,405 (32.2%) | 840 (31.8%) | 421 (33.1%) | 390 (30.1%) | 496 (35.2%) | 532 (34.3%) | 407 (34.0%) | 1,391 (29.9%) |
| Current | 833 (19.1%) | 466 (17.6%) | 194 (15.3%) | 230 (17.8%) | 205 (14.6%) | 310 (20.0%) | 221 (18.5%) | 957 (20.5%) |
| Missing | 72 (1.7%) | 80 (3.0%) | 20 (1.6%) | 8 (0.6%) | 61 (4.3%) | 28 (1.7%) | 27 (2.2%) | 135 (2.9%) |
| Years of theoretical education* |  |  |  |  |  |  |  |  |
| ≤10 years | 137 (3.1%) | 58 (2.2%) | 14 (1.1%) | 11 (0.9%) | 35 (2.5%) | 41 (2.6%) | 34 (2.9%) | 23 (0.5%) |
| 11-13 years | 1,374 (31.5%) | 488 (18.5%) | 296 (23.3%) | 249 (19.2%) | 345 (24.5%) | 454 (29.2%) | 295 (24.6%) | 1,437 (30.9%) |
| 14-17 years | 1,865 (42.8%) | 1,216 (46.0%) | 467 (36.7%) | 614 (47.4%) | 491 (34.8%) | 576 (37.1%) | 538 (44.9%) | 2,239 (48.1%) |
| ≥18 years | 586 (13.4%) | 666 (25.2%) | 366 (28.8%) | 258 (19.9%) | 419 (29.8%) | 374 (24.1%) | 240 (20.0%) | 578 (12.4%) |
| Missing | 400 (9.2%) | 215 (8.1%) | 128 (10.1%) | 164 (12.6%) | 119 (8.4%) | 108 (7.0%) | 91 (7.6%) | 380 (8.1%) |
| Known diabetes mellitus |  |  |  |  |  |  |  |  |
| No | 4,126 (94.6%) | 2,517 (95.2%) | 1,223 (96.2%) | 1,236 (95.4%) | 1,336 (94.8%) | 1,494 (96.2%) | 1,136 (94.8%) | 4,389 (94.2%) |
| Yes | 225 (5.2%) | 126 (4.8%) | 45 (3.5%) | 55 (4.2%) | 70 (5.0%) | 55 (3.5%) | 62 (5.2%) | 264 (5.7%) |
| Missing | 11 (0.25%) | 0 (0%) | 3 (0.2%) | 5 (0.4%) | 3 (0.2%) | 4 (0.25%) | 0 (0%) | 4 (0.1%) |
| Dental visit within the last 12 months |  |  |  |  |  |  |  |  |
| No | 485 (11.1%) | 292 (11.1%) | 122 (9.6%) | 109 (8.4%) | 132 (9.4%) | 155 (10.0%) | 103 (8.6%) | 315 (6.8%) |
| Yes | 2,584 (59.2%) | 1,785 (67.5%) | 888 (69.9%) | 865 (66.7%) | 920 (65.3%) | 982 (63.2%) | 820 (68.5%) | 2,893 (62.1%) |
| Missing | 1,293 (29.6%) | 566 (21.4%) | 261 (20.5%) | 322 (24.9%) | 357 (25.3%) | 416 (26.8%) | 275 (22.9%) | 1,449 (31.1%) |

Data are presented as mean ± standard deviation or N (%). * International Standard Classification of Education 97 (ISCED-97)

Supplemental Table 3. Overview on prevalence, severity, and extent of probing depths ≥6 mm using Level 2 data from the NAKO. Data are presented in total and stratified by sex, age, and study region.

|  |  |  | PD ≥6 mm | | | | |
| --- | --- | --- | --- | --- | --- | --- | --- |
|  | N total | N dentates | Prevalence, % * | Prevalence, % | Percentage of affected sites, % | Number of affected teeth | Percentage of affected teeth, % |
| Total | 18,389 | 18,216 | 6.0 (5.4-6.6) | 6.1 (5.5-6.7) | 0.7 (0.6-0.8) | 0.1 (0.1-0.1) | 1.2 (1.0-1.4) |
| By sex | | | | | | | |
| Female | 8,756 | 8,672 | 6.1 (5.2-7.2) | 6.1 (5.2-7.2) | 0.7 (0.5-0.8) | 0.1 (0.1-0.1) | 1.2 (0.9-1.4) |
| Male | 9,633 | 9,544 | 6.0 (5.2-6.9) | 6.0 (5.2-6.9) | 0.7 (0.6-0.9) | 0.1 (0.1-0.2) | 1.2 (1.0-1.5) |
| By age | | | | | | | |
| 19-29 years | 1,696 | 1,696 | 2.3 (1.4-3.7) | 2.3 (1.4-3.7) | 0.1 (0.1-0.2) | 0.0 (0.0-0.0) | 0.2 (0.1-0.3) |
| 30-39 years | 1,946 | 1,946 | 4.2 (2.8-6.4) | 4.2 (2.8-6.4) | 0.3 (0.2-0.4) | 0.1 (0.0-0.1) | 0.5 (0.3-0.8) |
| 40-49 years | 4,870 | 4,867 | 5.3 (4.2-6.8) | 5.3 (4.2-6.8) | 0.6 (0.4-0.9) | 0.1 (0.1-0.2) | 1.1 (0.6-1.6) |
| 50-59 years | 5,257 | 5,220 | 7.4 (6.3-8.6) | 7.5 (6.4-8.7) | 1.1 (0.8-1.3) | 0.2 (0.1-0.2) | 1.8 (1.4-2.2) |
| 60-74 years | 4,620 | 4,487 | 9.8 (8.4-11.4) | 10.4 (9.0-12.1) | 1.2 (1.0-1.5) | 0.2 (0.1-0.2) | 2.1 (1.7-2.6) |
| By study region | | | | | | | |
| Augsburg | 4,362 | 4,321 | 2.1 (1.3-3.2) | 2.1 (1.3-3.2) | 0.2 (0.1-0.3) | 0.0 (0.0-0.1) | 0.4 (0.1-0.6) |
| Mannheim | 2,643 | 2,620 | 4.1 (3.0-5.4) | 4.1 (3.1-5.5) | 0.5 (0.3-0.7) | 0.1 (0.0-0.1) | 0.6 (0.4-0.8) |
| Muenster | 1,271 | 1,263 | 4.4 (3.0-6.5) | 4.4 (3.0-6.6) | 0.4 (0.2-0.6) | 0.1 (0.0-0.1) | 0.5 (0.3-0.8) |
| Berlin North | 1,296 | 1,282 | 10.3 (7.2-14.6) | 10.6 (7.4-15.0) | 1.0 (0.6-1.4) | 0.2 (0.1-0.3) | 1.9 (1.2-2.6) |
| Hanover | 1,409 | 1,400 | 5.7 (3.6-8.9) | 5.7 (3.6-9.0) | 0.9 (0.3-1.5) | 0.2 (0.0-0.3) | 1.5 (0.4-2.7) |
| Hamburg | 1,553 | 1,535 | 5.2 (3.3-8.0) | 5.3 (3.4-8.2) | 0.4 (0.3-0.6) | 0.1 (0.1-0.1) | 0.8 (0.5-1.0) |
| Kiel | 1,198 | 1,188 | 4.3 (2.7-6.9) | 4.4 (2.8-6.9) | 0.8 (0.3-1.3) | 0.1 (0.1-0.2) | 1.1 (0.5-1.7) |
| Neubrandenburg | 4,657 | 4,607 | 10.8 (9.4-12.3) | 10.9 (9.6-12.5) | 1.3 (1.0-1.5) | 0.2 (0.2-0.2) | 2.4 (1.9-2.8) |

Data are presented as survey-weighted estimates (95% CI). * including edentulous individuals in the denominator (edentulous individuals were counted as non-prevalent individuals; information from Level 1 tooth counts was considered to define edentulism)

Supplemental Table 4. Data on edentulism and number of teeth (including third molars) in participants aged 65-74 in the German National Cohort (NAKO; Level 1), the Fifth German Oral Health Study (DMS V), and the Study of Health in Pomerania (SHIP-TREND-0).

|  | N | Edentulism, % | Number of teeth | N | Number of teeth  in dentates |
| --- | --- | --- | --- | --- | --- |
| *NAKO* | | | | | |
| Augsburg/ Regensburg | 3,833 | 4.9 (3.5-6.7) | 20.3 (19.7-20.9) | 3,659 | 21.3 (20.8-21.8) |
| Mannheim/ Freiburg/ Saarbrücken | 3,653 | 6.5 (5.1-8.2) | 19.7 (19.1-20.3) | 3,491 | 21.0 (20.5-21.6) |
| Essen/ Muenster/ Düsseldorf | 3,701 | 7.5 (5.9-9.5) | 19.7 (19.2-20.3) | 3,538 | 21.3 (20.9-21.8) |
| Halle/ Leipzig | 2,237 | 7.1 (5.0-10.1) | 18.6 (17.9-19.3) | 2,119 | 20.0 (19.4-20.5) |
| Berlin South/ Central/ North | 4,224 | 6.9 (5.4-8.7) | 20.0 (19.5-20.5) | 4,052 | 21.5 (21.1-21.9) |
| Hanover/ Hamburg/ Bremen | 4,178 | 7.2 (5.8-8.9) | 19.7 (19.1-20.2) | 4,014 | 21.2 (20.7-21.6) |
| Kiel | 1,189 | 5.2 (3.3-8.2) | 20.1 (19.2-21.1) | 1,145 | 21.2 (20.4-22.1) |
| Neubrandenburg | 2,245 | 6.0 (4.7-7-6) | 17.5 (16.9-18.0) | 2,131 | 18.6 (18.0-19.1) |
| *DMS V* | | | | | |
| North-east/East: Mecklenburg-Western Pomerania/ Brandenburg/ Berlin/ Saxony-Anhalt/ Saxony/ Thuringia | 378 | 13.4 (9.6-18.2) | 16.4 (15.1-17.6) | 336 | 18.9 (17.8-19.9) |
| North-west: Schleswig-Holstein/ Hamburg/ Bremen/ Lower Saxony | 146 | 5.5 (2.7-9.5) | 18.8 (17.4-20.1) | 137 | 19.9 (18.6-21.1) |
| Mid-west: North Rhine-Westphalia | 145 | 19.2 (14.3-24.7) | 16.2 (14.8-17.6) | 119 | 20.0 (18.9-21.1) |
| South-west: Baden-Wuerttemberg/ Saarland/Hesse/ Rhineland-Palatinate | 240 | 14.5 (10.6-19.0) | 17.7 (16.6-18.9) | 208 | 20.7 (19.8-21.6) |
| South: Bavaria | 133 | 5.5 (2.9-10.3) | 18.6 (17.4-19.8) | 125 | 19.7 (18.6-20.7) |
| *SHIP-TREND-0* | | | | | |
| Western Pomerania | 756 | 15.7 (12.9-18.5) | 13.2 (12.5-13.9) | 646 | 15.7 (15.0-16.3) |

Data are presented as survey-weighted estimates (95% CI).

Supplemental Table 5. Characteristics of participants aged 65-74 in the German National Cohort (NAKO; Level 1), the Fifth German Oral Health Study (DMS V), and the Study of Health in Pomerania (SHIP-TREND-0).

|  | NAKO  (2014-2019) | DMS V (2014) | SHIP-TREND-0  (2008-2012) |
| --- | --- | --- | --- |
| N | 25,260 | 1024 | 756 |
| Age, years | 67.4±1.8 | 69.4±3.0 | 69.3±2.8 |
| Sex |  |  |  |
| Male | 12,876 (51.0%) | 490 (47.0%) | 390 (51.6%) |
| Female | 12,384 (49.0%) | 552 (53.0%) | 366 (48.4%) |
| Smoking status |  |  |  |
| Never | 10,533 (41.7%) | 545 (52.3%) | 351 (46.4%) |
| Former | 10,233 (40.5%) | 369 (35.4%) | 333 (44.05%) |
| Current | 2,670 (10.6%) | 123 (11.8%) | 66 (8.7%) |
| Missing | 1,824 (7.2%) | 5 (0.5%) | 6 (0.8%) |
| Years of theoretical education* |  |  |  |
| ≤10 years | 881 (3.5%) | - | - |
| 11–13 years | 6,488 (25.7%) | - | - |
| 14–17 years | 11,069 (43.8%) | - | - |
| ≥18 years | 5,037 (19.9%) | - | - |
| Missing | 1,785 (7.1%) | - | - |
| School education |  |  |  |
| <10 years | - | 469 (45.0%) | 416 (55.0%) |
| 10 years | - | 255 (24.6%) | 178 (23.5%) |
| >10 years | - | 260 (25.0%) | 159 (21.0%) |
| Missing | - | 58 (5.6%) | 3 (0.4%) |
| Known diabetes mellitus |  |  |  |
| No | 21,740 (86.1%) | 873 (83.8%) | 595 (78.7%) |
| Yes | 3,446 (13.6%) | 169 (16.2%) | 159 (21.0%) |
| Missing | 74 (0.3%) | 0 (0%) | 2 (0.3%) |

Data are presented as mean ± standard deviation or N (%). * International Standard Classification of Education 97 (ISCED-97)

Supplemental Figure 1. Distribution of Level 1 and Level 2 study centres within Germany.

Supplemental Figure 2. A) Number of missing teeth (including edentulous persons) according to age within Level 1 study regions and B) mean probing depth according to age within Level 2 study regions of the NAKO.

Unweighted numbers of participants within study regions and age groups are: Augsburg: 2814/ 2901/ 7541/ 7420/ 7115; Mannheim: 2725/ 2985/ 7115/ 7136/ 7127; Muenster: 2969/ 3101/ 7351/ 7462/ 7306; Berlin North: 1739/ 1759/ 4679/ 4596/ 4365; Hanover: 3000/ 3027/ 7929/ 7849/ 7787; Hamburg: 2924/ 3022/ 7399/ 7609/ 7658; Kiel: 877/ 988/ 2275/ 2396/ 2325; Neubrandenburg: 1555/ 2149/ 4293/ 4824/ 4779.

Supplemental Figure 3. Correlation between and response for Level 1 study regions.
